# Long-Term Fatigue and Cognitive Disorders in Breast Cancer Survivors

**DOI:** 10.3390/cancers11121896

**Published:** 2019-11-28

**Authors:** Florence Joly, Marie Lange, Melanie Dos Santos, Ines Vaz-Luis, Antonio Di Meglio

**Affiliations:** 1 Clinical Research Department, Centre François Baclesse, 14000 Caen, France; m.lange@baclesse.unicancer.fr (M.L.); m.dossantos@baclesse.unicancer.fr (M.D.S.); 2 INSERM U1086, ANTICIPE, Normandie Univ, UNICAEN, 14000 Caen, France; 3 Cancer and Cognition Platform, Ligue Nationale Contre le Cancer, 14000 Caen, France; 4 University Hospital of Caen, 14000 Caen, France; 5 INSERM Unit 981, Gustave Roussy, 94800 Villejuif, France; INES-MARIA.VAZ-DUARTE-LUIS@gustaveroussy.fr (I.V.-L.); Antonio.DI-MEGLIO@gustaveroussy.fr (A.D.M.)

**Keywords:** breast cancer, survivors, fatigue, cognition, late effects

## Abstract

Survivors of early-stage breast cancer may report treatment-related side effects that persist for several years after the end of primary treatment. Among these, fatigue and cognitive disorders are frequent complaints and can negatively impact quality of life. Cancer-related fatigue is a very prevalent and distressing long-term side effect among breast cancer survivors that typically improves after completion of treatment, although many patients report severe fatigue several years post-treatment. Cognitive disorders are also common among survivors of breast cancer, especially if treated with chemotherapy. These symptoms are usually mild-to-moderate and often transient. Cognitive recovery is frequently observed within months or a few years after completion of chemotherapy or endocrine therapy. However, some breast cancer survivors may have persistent cognitive difficulties. Several types of interventions have proved to be beneficial in reducing cancer-related fatigue and cognitive difficulties. Most of these interventions for cancer-related fatigue are thought to be effective by reducing inflammation or disrupting pro-inflammatory circuits. Further studies are needed on cognitive management that has showed promising results. This narrative review summarizes the state of the art regarding long-term fatigue and cognitive disorders in patients with early breast cancer, describing prevalence, impact, pathophysiology, and risk factors, and focusing on available interventions.

## 1. Introduction

Breast cancer is one of the most common cancers worldwide, with almost 80% of patients expecting long-term disease-free survival. As cure rates have substantially improved, there is now an increased awareness of long-term side effects of cancer treatment [1,2,3]. Cancer-related fatigue and cognitive complaints are common symptoms after breast cancer diagnosis and treatment. Both of these symptoms can persist for years after the end of primary treatment and result in substantial adverse physical, psychosocial, and socio-economic consequences [4,5,6,7,8,9,10].

This review presents an update of the state of the art regarding long-term fatigue and cognitive disorders in patients with early breast cancer, describing prevalence, impact, pathophysiology, and risk factors, and focuses on available interventions. We performed a narrative review of studies published up until September 2019. We restricted our search to papers in the English language, and included observational studies, randomized controlled trials, as well as meta-analyses and systematic reviews.

## 2. Long-Term Cancer-Related Fatigue in Breast Cancer Survivors: Prevalence, Trajectory, and Impact

### 2.1. Assessment and Prevalence

Cancer-related fatigue is an extremely prevalent long-term side effect among breast cancer survivors [11], defined as “a distressing, persistent, subjective sense of physical, emotional, and/or cognitive tiredness or exhaustion related to cancer and/or cancer treatment that is not proportional to recent activity and interferes with usual functioning” [12]. Cancer-related fatigue can have distinct physical, cognitive, and emotional dimensions, and seems to be more severe, persistent, and debilitating than normal fatigue. Several studies have suggested that the intensity and duration of fatigue experienced by cancer patients and survivors is significantly greater than that of individuals without a history of cancer [13,14]. 

Because fatigue is a subjective experience, the gold standard to evaluate cancer-related fatigue is self-reporting. A practical way of evaluating fatigue is to ask patients to evaluate their fatigue using a scale ranging from 0–10, which classifies fatigue as mild if patients score 1–3, moderate if 4–6, and severe if 7–10. In addition, cancer-related fatigue can be evaluated by a number of validated questionnaires that vary from single-item to multi-dimensional instruments, which can characterize different aspects of fatigue, including severity, duration, interference, and dimensions. Some of the validated instruments to assess cancer-related fatigue are listed in Table 1 [5,6,8]. Of note, the great heterogeneity that exists in instruments to evaluate fatigue is often reported as a major limitation in the available literature. 

Overall, during the phase of active treatment, the vast majority of patients experience some fatigue, with 30–60% categorizing their fatigue as moderate or severe, depending on patient population, patterns of treatment, and instrument of evaluation. Although fatigue typically improves after completion of primary treatment for breast cancer (surgery, radiation, and/or chemotherapy), around 30% of patients continue to experience severe fatigue in the year after treatment, and 20% of patients still report severe fatigue at 10 years post-treatment [5,6,8]. Recently, investigators from the Mind-Body Study characterized five longitudinal trajectories of fatigue after breast cancer treatment over time up to 6 years after treatment among 191 breast cancer patients [15]. The identified groups included: (1) High fatigue (11%), characterized by persistently elevated fatigue; (2) recovery (28%), characterized by initially high but then decreasing fatigue; (3) late (17%), characterized by initially low but gradually increasing fatigue; (4) low (34%), characterized by initially low and gradually decreasing fatigue; and (5) very low (10%), characterized by persistently stable and low fatigue.

### 2.2. Impact of Cancer-Related Fatigue after Breast Cancer

Data suggest that fatigue can affect patients’ social and work lives (e.g., a small minority of patients will be so debilitated by fatigue that they will be unable to work or regain their premorbid level of health) [11]. Fatigue can substantially impact breast cancer survivors’ ability to respond to work demands, in particular in the case of heavy workload or work-related stress. In addition, fatigue may have substantial negative consequences on quality of life and other daily activities. Cancer-related fatigue is often associated with severe emotional and role dysfunction and with a lack of life meaning [6,7,16]. Reports show that patients consider fatigue as a highly distressing symptom, which is often being referred to as more difficult to cope with than vomiting or pain [17]. Nevertheless, despite the prevalence and impact of this symptom, data also suggest that fatigue is highly underreported by patients and remains an often unaddressed issue by oncology providers. 

### 2.3. Risk Factors and Mechanisms of Long-Term Cancer-Related Fatigue after Treatments for Breast Cancer

Fatigue is a complex and multidimensional symptom. A number of studies have investigated and proposed a variety of risk factors and mechanisms of onset for long-term cancer-related fatigue [8,18]. 

#### 2.3.1. Risk Factors of Cancer-Related Fatigue

Demographic and contextual factors such as young age, being single, having a low income, as well as medical and psychosocial factors, including multiple comorbidities, physical inactivity, elevated body mass index, pre-treatment fatigue, depression, early stress, sleep disturbance, dysfunctional coping, and loneliness, have all been associated with increased risk of fatigue [18]. In this setting, pre-treatment fatigue has been identified as the most consistent predictor of post-treatment fatigue [11]. In addition, tumor and treatment factors can also impact risk of cancer-related fatigue. Patients with higher cancer stage are at increased risk of fatigue, as well as those treated with combination modalities that include surgery and radiotherapy with or without hormonotherapy. On the contrary, patients treated only with local strategies such as surgery with or without radiotherapy are at lower risk of severe fatigue [5,6,8]. More recently, some studies also identified genetic risk factors that were linked to increased risk of developing cancer-related fatigue. Most of such studies focused on genes associated with inflammation using a candidate-gene approach. Among these, polymorphisms in TNFa, IL6, and IL1-b were associated with fatigue after breast cancer treatment completion. However, these results were not consistently replicated across studies. These polymorphisms have been associated with fatigue also in non-cancer populations, suggesting that they may play a role in the biology underlying fatigue in a more generalizable fashion [19,20]. 

#### 2.3.2. Mechanisms of Cancer-Related Fatigue 

Several mechanisms have been proposed to be linked with cancer-related fatigue. These include, among others, inflammation, hypothalamic pituitary adrenal (HPA) dysfunction, five hydroxyl tryptophan (5-HT) dysregulation, alterations in the autonomic nervous system, alterations in adenosine triphosphate and muscle metabolism, changes in leukocyte subsets, reactivation of latent herpes viruses, anemia, and down-regulation of genes with response elements for the glucocorticoid receptor [21,22,23,24,25]. The lack of longitudinal studies in this setting makes it difficult to interpret whether or not all of the cited biological changes are only driven by cancer and cancer treatment or might also be present before cancer diagnosis. 

The most commonly explored biological and mechanistic model for cancer-related fatigue involves inflammation and associated neurologic activation. Several studies have documented increased inflammation markers post breast cancer treatment, including elevation of IL-1β, TNF-α, IL-6, soluble TNF receptor type II (sTNF-RII), IL-1 receptor antagonist (IL-1RA), soluble IL-6 receptor (sIL-6R), and C-reactive protein (CRP), with the hypothesis that peripheral inflammatory cytokines may promote signals to the central nervous system that generate symptoms of fatigue [11,26,27,28]. This is consistent with studies performed among otherwise healthy individuals where sub-clinical levels of inflammatory markers are associated with the development of fatigue [11,29,30,31]. These data can intersect with evidence highlighting the relation of fatigue and alterations of the HPA axis and autonomous nervous system. Indeed, both of these systems have important anti-inflammatory effects since they interfere with cytokine production, or, such as in the case of the HPA axis, via glucocorticoid production or decreased sensitivity of glucocorticoid receptor [11,21,27,32,33,34,35]. Another biological process that may influence fatigue includes alterations of the immune system, which can also be linked with increased inflammation [11,21,27,32,33,34]. 

## 3. Interventions to Reduce Cancer-Related Fatigue

Several types of interventions have proved to be beneficial in reducing cancer-related fatigue among survivors of breast cancer, although no clear superiority of one over the other has been demonstrated, and standards are lacking to select the most appropriate intervention for individual patients. Most of these interventions are thought to be effective because they reduce inflammation or disrupt pro-inflammatory circuits [11]. Leading cancer societies, including the National Comprehensive Cancer Network (NCCN), the Oncology Nursing Society (ONS), and the American Society of Clinical Oncology (ASCO), have released several recommendations about interventions that can be offered to patients to reduce cancer-related fatigue. Most of these recommendations are based on studies that largely included breast cancer survivors.

Assessment and correction of treatable contributing factors, including nutrition status (e.g., vitamin status, weight/caloric intake changes, and fluid electrolyte imbalances), are strongly encouraged before considering interventions to reduce fatigue.

A summary of the proposed interventions for cancer-related fatigue is presented in Table 2.

### 3.1. Counseling and Education on Cancer-Related Fatigue

In a Cochrane review of randomized trials among cancer patients, educational interventions reduced cancer-related fatigue and its interference with daily life [36]. Therefore, in general, all patients should be educated on cancer-related fatigue (e.g., how it differs from normal fatigue, what are the contributing factors, and how to self-monitor fatigue levels, and how to manage cancer-related fatigue). This is particularly relevant for patients that receive treatments that have been linked with increased risk of cancer-related fatigue, including radiation and chemotherapy. Cancer survivors should always be made aware of providers and facilities they can refer to in order to engage in programs that may help reduce cancer-related fatigue, and this includes distant-based alternatives. Indeed, studies have also shown that counseling can also be effectively delivered remotely via tele-health/internet to patients that are not in the active phase of treatment [37]. 

### 3.2. Physical Activity Interventions

Among non-pharmacologic interventions, the strongest and most consistent evidence supports the effectiveness and safety of physical activity interventions in reducing cancer-related fatigue. A large number of studies have evaluated the impact of exercise on fatigue in the active treatment or immediate post-treatment settings, with fewer data available on longer-term cancer-related fatigue. A meta-analysis of 27 exercise intervention trials showed that exercise training among patients with various types of cancer led to a significant reduction in fatigue. Exercise significantly reduced cancer-related fatigue with a mean effect size of 0.32 (95% Confidence Interval [CI] 0.21–0.43) during cancer treatment and 0.38 (95% CI 0.21–0.54) following treatment completion. In the post-treatment setting, longer time between treatment completion and exercise intervention initiation and shorter exercise program length were linked to larger improvements in fatigue score [38]. In another meta-analysis of 72 studies among patients with either solid or hematological or mixed malignancies, exercise reduced cancer-related fatigue compared to control, with a moderate effect (standardized mean difference (SMD), −0.45; 95% CI −0.57 to −0.32) [39]. Finally, a Cochrane meta-analysis conducted including 56 randomized trials, 36 of which enrolled patients in the phase of active cancer treatment, demonstrated that there was both a decrease in fatigue from baseline to 12 weeks after baseline and better fatigue scores at 12 weeks after baseline, favoring patients in the exercise intervention arms (SMD, −0.38; 95% CI −0.57 to −0.18 and SMD −0.73; 95% CI −1.14 to −0.31, respectively) [40]. 

Based on these data, the National Comprehensive Cancer Network (NCCN) diffused guidelines supporting physical activity intervention as a Category 1 recommendation for the treatment of cancer-related fatigue, particularly among patients who have completed treatment [12]. Although there is still no evidence to recommend a specific amount of physical activity, all patients are encouraged to engage in at least moderate levels of physical activity after cancer treatment to reduce cancer-related fatigue, as reported in specific guidelines published by ASCO. A minimum of 150 minutes of moderate aerobic exercise per week is sufficient to meet the threshold of the recommended level, and this may include whatever form of aerobic training (e.g., fast walking, cycling, or swimming) in addition to 2–3 sessions of strength training (e.g., including weight lifting), unless specific contraindications exist [41]. 

Nevertheless, exercise interventions to reduce cancer-related fatigue should be tailored to the individual patient, based on factors including age, gender, and baseline level of physical fitness. Specifically, the choice of the most appropriate physical activity intervention to offer should account for patients’ medical history and comorbidities (including neuropathy, cardiomyopathy, or other long-term effects of therapy), as well as for physical limitations, risk of injury, and other safety concerns, particularly in the case of structural bone problems or blood test abnormalities such as moderate-to-severe anemia or thrombocytopenia [12]. However, several exercise programs exist that are suitable and considered safe for most cancer survivors, particularly those programs that are walking-based. 

Some evidence has demonstrated that yoga interventions are able to reduce fatigue during cancer treatment. The majority of tested interventions were conducted among women with breast cancer undergoing chemotherapy. For example, a small randomized clinical trial included 60 patients with breast cancer during active adjuvant chemotherapy, and showed that 8 weeks of Anusara yoga sessions twice per week improved fatigue [42]. Nevertheless, evidence on the relationship between engaging in yoga practices and reduction in cancer-related fatigue is not always consistent. A larger trial of 352 women with early-stage breast cancer receiving chemotherapy failed to demonstrate that participation in a Tibetan yoga program during active chemotherapy and over the following six months significantly reduces cancer-related fatigue compared to a stretching program or usual care; despite this, the yoga program was associated with better sleep outcomes in exploratory analyses, and an amelioration of such sleep parameters may in turn translate into improved cancer-related fatigue [43]. 

### 3.3. Other Physical-Based Therapies and Mind–Body Interventions

There are varying degrees of evidence strength about the effectiveness of interventions such as massage therapy, acupuncture, music therapy, mindfulness meditation and relaxation, reiki, and qigong on reducing cancer-related fatigue. Most of the studies we present below included a substantial proportion of breast cancer survivors evaluated in the post-treatment setting.

A meta-analysis of randomized controlled trials included 18 studies and compared the impact of regular use of massage therapy versus control on fatigue symptoms in patients with breast cancer. Regular massage therapy determined greater reduction in fatigue compared to control (SMD, −0.61; 95% CI −1.09 to −0.13) [44]. 

Acupuncture interventions were also tested among the cancer population to evaluate their benefits in reducing cancer-related fatigue, including in patients during and after chemotherapy treatment. These trials have been generally small and some have reported positive effects of acupuncture on fatigue [45]. One such trial compared infrared laser moxibustion (i.e., acupuncture combined with burning of herb moxa on or near the skin by acupoints) with sham laser moxibustion, showing improved fatigue among the group treated with the former treatment [46]. 

Finally, some other interventions may also offer some benefit to mitigate cancer-related fatigue, including music therapy, meditation and relaxation, reiki, and qigong [47,48]. However, available evidence is not yet very robust, and often limited by small sample study size, limited power, suboptimal adherence in interventional arms of these trials, and scarce data that could inform practice in specific settings, particularly in the long-term post-treatment setting. Importantly, ASCO recommends that if physical or mind–body interventions are prescribed, patients should be referred to practitioners that specialize in cancer and are trained and used to protocols empirically validated in cancer survivors [41]. 

### 3.4. Psychosocial Interventions

Cognitive-behavioral therapy and psycho-educational/educational therapies can be beneficial in reducing cancer-related fatigue, as demonstrated by some randomized trials and meta-analyses. The rationale that prompted the utilization of psychosocial interventions to reduce fatigue builds on the strong correlation that exist between emotional distress and cancer-related fatigue. Studies that evaluated psychosocial interventions aimed at targeting biologic mechanisms such as neurotransmitter deregulation, vagal activation, or circadian rhythm dysfunction to be able to modulate cancer-related fatigue, although little is known regarding the precise mechanisms that mediate this relationship. In fact, most of the studied psychosocial interventions have tried to improve coping mechanisms, through which patients would be more able to deal with fatigue rather than disrupting the underlying biological and mechanistic process of onset of fatigue [12,41]. 

Most of the available evidence comes from studies in a diverse population of cancer survivors, not only breast cancer patients. In a study that targeted dysfunctional thoughts about fatigue, cognitive-behavioral therapy determined a significant reduction in fatigue that was also sustained two years after completion of the program [49]. Analogously, an Internet-based tailored education program was evaluated among disease-free cancer survivors who had completed primary cancer treatment within the past 24 months, and that complained of moderate-to-severe cancer-related fatigue, and the trial demonstrated that the intervention group had greater improvements in fatigue compared to control [50]. Finally, in a combined and comprehensive systematic and meta-analytic review of non-pharmacological interventions in adults with cancer that looked both at exercise and psychological interventions, those that were based on restorative approaches, supportive-expressive, and cognitive-behavioral psychosocial therapy showed potential to improve cancer-related fatigue [51]. 

### 3.5. Pharmacologic Interventions

Evidence regarding the use of pharmacologic interventions in reducing long-term fatigue in patients with early breast cancer is still limited. Psychostimulants (e.g., methylphenidate) and other wakefulness agents (e.g., modafinil, a non-amphetamine psychostimulant) were shown to be effective in reducing cancer-related fatigue among patients with advanced disease or those undergoing active cancer treatment [52,53]. However, whether these drugs are effective for patients who are disease-free after active treatment is still an open question. In addition, safety concerns and only marginal improvements in cancer-related fatigue observed with some of these compounds limit the ability to recommend their utilization as a primary choice for cancer-related fatigue.

The use of supplements has also been investigated, and use of ginseng and vitamin D has been linked to some degree with improved cancer-related fatigue [54]. However, evidence is still heterogeneous and weak.

## 4. Long-Term Cognitive Disorders in Breast Cancer Survivors

Cancer-related cognitive impairment (CRCI) has been described after chemotherapy in breast cancer women (“*chemobrain*”) and is characterized by impairment of memory, executive functions, attention, and processing speed [55,56].

Cognitive functioning is usually distinct between objective cognition, assessed with neuropsychological tests, and subjective cognition, also called cognitive complaints, reported by the patients and assessed with self-report questionnaires. Cognitive complaints were reported by ≥50% of breast cancer patients after chemotherapy; however, only 15–25% had objective cognitive decline [57]. Cognitive complaints, often reported by patients with poor emotional status or reported fatigue, are not always linked to objective cognitive disorders [58]. Indeed, many studies showed an association between reported cognitive complaints and psychological factors such as depression [59,60,61] or anxiety [61,62], as well as with fatigue and sleep difficulties [63,64]. Nevertheless, although there is a lack of correlation between objective and subjective cognitive problems, patients’ perceptions of cognitive disorders represent an important survivorship issue because of their significant effect on quality of life.

Although CRCI is a well-described phenomenon and awareness of such a problem has recently increased among the medical community, only 37% of breast cancer survivors with cognitive difficulties report to have discussed these concerns with a healthcare provider [65], and most healthcare professionals declare to be uncertain about how to manage CRCI [66].

### 4.1. Chemotherapy and Cognitive Impairment

After chemotherapy, objective cognitive difficulties are usually mild-to-moderate and are often transient. Cognitive decline shortly after chemotherapy was reported by some breast cancer patients, followed by partial recovery one year after treatment completion [67]. Patients treated with anthracycline-containing chemotherapy regimens are more at risk to present decline of verbal memory performances compared to those not treated with anthracyclines [68]. However, reduced cognitive performance and white matter alterations observed on imaging were found to recover in a group of breast cancer survivors 3–4 years after chemotherapy [69].

Nevertheless, some breast cancer survivors had worse performances on cognitive tests compared to healthy controls for up to 20 years after the end of adjuvant chemotherapy [70], including fine motor function deficits [71]. Furthermore, an imaging study showed that time since cancer treatment was inversely associated with lower global and focal white matter integrity [72]. Decreased responsiveness of brain regions related to memory encoding and executive functioning was also observed 10 years after chemotherapy in breast cancer survivors [73]. In addition, a dose-dependent effect of adjuvant chemotherapy was also suggested on grey matter volume one decade after treatment [74].

Some longitudinal studies have focused on cognitive complaints in breast cancer survivors, suggesting partial recovery from cognitive complaints six months after chemotherapy, but without returning to pretreatment level [61]. Heterogeneous cognitive complaint trajectories have been described 15 months post-chemotherapy: 53% of breast cancer survivors did not report cognitive complaints, 30% showed significant cognitive alterations, 16% had acute cognitive changes, and 11% persistent difficulties [75]. Specifically, in older breast cancer survivors followed up annually for 7 years, the majority reported good long-term cognition and only 6% who received chemotherapy presented accelerated cognitive decline [76].

### 4.2. Endocrine Therapy and Cognitive Impairment

In addition to the impact of chemotherapy on cognition, endocrine therapies could also affect cognition. In a recent meta-analysis, endocrine therapy was associated with impaired performance on some neuropsychological tests, especially those which assessed verbal learning/memory domain [77]. Difference of the impact on cognition of tamoxifen or aromatase inhibitors is still debated. However, cognitive impairment has been shown more frequently with non-steroid aromatase inhibitors [55]. Up to 18 months after the start of treatment, breast cancer survivors who received anastrozole had lower executive function scores than healthy controls [78]. In the same study, 12–18 months after initiation of therapy, women being treated with anastrozole alone exhibited decrease in working memory and concentration. Verbal memory also appeared to be affected one year post-treatment [79], whereas no detrimental effect of endocrine therapy on cognition was shown 6 years after the start of treatment [80].

### 4.3. Impact of Cognitive Impairment

Cognitive dysfunction may substantially impact quality of life and social function. An example of such negative impact is the ability of return to work after cancer treatment. Indeed, five years post-diagnosis, in addition to fatigue and psychological problems, cognitive disorders are related with difficulties in returning to work in breast cancer survivors [81]. Furthermore, cognitive complaints were shown to be associated with poorer work ability, work performance, and work productivity [82]. Stressing the relevance of this concern from a patient’s perspectives, some reports have shown that breast cancer survivors with CRCI express the need of appropriate support to maintain their quality of life and be facilitated to return to work [83,84].

### 4.4. Mechanisms of Long-Term Cognitive Disorders After Treatment for Breast Cancer 

The mechanisms involved in CRCI are complex and still not fully understood [55]. 

In addition to the impact of cancer and cancer treatments, the risk of persistent cognitive impairment is linked to a patient’s characteristics, such as lifestyle, comorbidities, physiological, psychological, and genetic parameters [85]. Individual factors induce some vulnerabilities for long-term cognitive impairment in breast cancer patients: The physiological age associated with some comorbidities (e.g., vascular disease, diabetes), low cognitive reserve, acceleration of shortened telomere length, and some psychosocial situations (e.g., maladaptive patterns of coping, post-traumatic stress, depression, low socioeconomic status, education) [86,87,88].

Neuroimaging studies, including structural and functional magnetic resonance imaging, have contributed to elucidating the mechanisms involved in long-term persisting cognitive decline. Some breast cancer survivors treated with chemotherapy presented brain gray volume loss, with a predominantly prefrontal and frontal cortex hypo-activation, white matter microstructural disruption, reduced gray matter density, and impaired cerebral blood flow [89,90,91]. Some other studies showed decreased network connectivity particularly in the frontal and temporal regions of breast cancer patients, even 10 years after chemotherapy [91,92]. Additionally, hippocampal structures are involved in different domains of cognitive impairment [93]. In some patients with cognitive difficulties, activation of compensatory mechanisms has been shown with the involvement of additional brain areas and the creation of new neural connections [94]. In a study of long-term breast cancer survivors treated with chemotherapy, patients who had greater memory dysfunction had more oxidative DNA damages due to oxidative stress and reduced gray matter density in comparison to controls [95]. 

Recent results have demonstrated a direct cerebral damage due to systemic anticancer treatments, but have also suggested a role for the dysregulation of immune system, inflammatory mediators, hormonal changes, and genetic predisposition as potential mechanisms of persistent cognitive decline [96]. 

Some chemotherapy drugs used in breast cancer, such as 5-fluorouracil, are able to cross the blood–brain barrier and have a direct neurotoxic effect on the brain with damage to neurons or glial cells, ischemic vascular damage, and a reduced number of hippocampal proliferating cells [97,98]. Inflammation has been suggested to play a critical role in long-term cognitive disorders with a strong association between blood cell-based inflammatory markers and cognitive performance in breast cancer survivors, even 20 years and over after cessation of chemotherapy [99]. High levels of CRP and cytokines (mainly IL6, IL1ß, and sTNF-RII) have been also reported in others studies among breast cancer survivors with cognitive impairment that suggested a dysregulation of the immune system as a possible mechanism of onset of cognitive decline [100,101]. The cortisol and hypothalamic pituitary adrenal axis has also been hypothesized to play a role in persistent cognitive impairment in breast cancer, associated with fatigue [34]. An elevation of circulating corticosterone is considered to be associated with memory impairment [102]. The negative impact of glucocorticoid levels on cognition is correlated with concomitant reductions in the volume of the hippocampus and with deficits in neurogenesis and synaptic plasticity [103]. Finally, genetic predispositions (such as Apolipoprotein Ee4, some polymorphisms of Catechol-O-Methyltransferase or Brain-derived Neurotropic Factor) have been shown to be associated with increased risk for cognitive disorders in survivorship [104,105]. 

## 5. Interventions for Cognitive Impairment after Breast Cancer Treatment

Supportive care of CRCI after treatments remains an emerging area of research. Despite the high demand of management by patients [106], currently there is no preventive measure and no medication that has clearly been established to improve or manage CRCI [107]. A summary of the proposed interventions for CRCI is presented in Table 3. Non-pharmacologic interventions include mainly cognitive rehabilitation and physical activity or relaxation programs.

Recently, guidelines for the assessment and management of long-term side-effects, including cognitive impairment, have been published by the American Cancer Society/ASCO Breast Cancer Survivorship Care. They recommend that primary care clinicians should ask patients if they are experiencing cognitive impairment and should assess whether factors contribute to their cognitive difficulties. In addition, they should treat optimally when possible and refer patients with signs of cognitive disorders for cognitive assessment and rehabilitation, including, if available, group cognitive training [2].

### 5.1. Cognitive Rehabilitation

Randomized controlled trials investigated the impact of cognitive rehabilitation program on cognitive impairment in breast cancer patients, with a positive impact on cognitive complaint and Quality of life. Von ah et al. evaluated, among 82 breast cancer survivors, 6–8 weeks of memory or processing intervention, with improvements in perceived cognitive functioning, symptom distress, and Quality of life [108]. Ercoli et al. demonstrated improvements in self-reported cognitive complaints [109]. Damholdt et al. evaluated 6-week web-based cognitive rehabilitation among 157 breast cancer survivors, with working memory as primary outcome measured by the Paced Auditory Serial Addition Test, with no significant difference found [110]. Bray et al. demonstrated improvements in cognitive complaints and symptom distress among 242 cancer patients, mostly with breast cancer (89%), with cognitive complaints after chemotherapy, randomly assigned to 15-week computer-assisted cognitive rehabilitation or control group [111]. No significant difference was found in objective cognitive assessment. Mihuta et al. also found a positive impact on perceived cognitive impairments in 4-week cognitive rehabilitation [112].

### 5.2. Physical Activity or Relaxation Program Interventions

Physical activity has already proven its worth for age-related cognitive decline [123]. However, few studies have evaluated its benefit on CRCI in the breast cancer survivors. Campbell et al. evaluated a randomized controlled trial of 24-week aerobic exercise intervention compared to usual lifestyle control among 19 women with self-reported cognitive dysfunction following chemotherapy for breast cancer [113]. Exercise intervention improved processing speed, but not self-reported cognitive function. Hartman et al. conducted a randomized controlled trial with 87 breast cancer survivors randomly assigned to a 12-week physical activity or control group [114]. An improvement on processing speed was also found, but only for patients diagnosed within the past 2 years, and no difference was demonstrated on self-reported cognition. Northey et al. conducted a 3-arm trial with 17 patients to compare high-intensity interval training, moderate-intensity continuous training, and wait-list control [115]. No significant difference was observed in the cognitive and cerebrovascular outcomes. Yoga was also evaluated in a few studies. Derry et al. conducted a large randomized controlled trial, with 200 breast cancer survivors reporting cognitive complaints, randomized to a 12-week twice-weekly yoga intervention or a wait-list. Cognitive complaints did not differ significantly between groups immediately post-intervention. However, at the 3-month follow-up visit, yoga participants reported significantly fewer cognitive complaints than wait-list participants [116]. Myers et al. evaluated in a 3-arm randomized controlled trial the impact of qigong, which is a mindfulness-based form of exercise originating from Chinese medicine, on cognitive function for breast cancer survivors with complaints [117]. Fifty patients were randomly assigned to qigong or gentle exercise or survivorship support. The qigong group showed significant improvements in cognitive complaints, processing speed, and distress compared to the two other arms.

### 5.3. Pharmacologic Interventions

Pharmacologic interventions include psychostimulants, with some studies evaluating particularly methylphenidate, a psychostimulant dopamine agonist, which is better known for the management of cancer-related fatigue [124,125]. No evidence of improvement in cognitive impairment was showed with this drug in two randomized controlled trials with cancer patients treated with chemotherapy compared with placebo [118,119]. Moreover, a higher rate of grade 3/4 side effects was reported in the experimental arm. Another psychostimulant, the modafinil, used in the treatment of narcolepsy, was also evaluated in an open-label Phase I/II study involving 82 breast cancer patients, with no significant effect on cognitive outcomes compared to placebo [120]. A recent clinical trial evaluated donepezil, an anti-Alzheimer’s medication compared to placebo among 62 breast cancer survivors with self-reported cognitive dysfunction 1–5 years following adjuvant chemotherapy [121]. After 24 weeks of intervention, donepezil showed improvement in memory scores, but not in subjective cognitive function or Quality of life. The impact of erythropoietin was also studied, with no robust evidence for a positive impact on CRCI [122].

## 6. Conclusions—Perspectives

Improved cure rates for survivors of early-stage breast cancer have contributed to shift the attention to long-term downstream effects of cancer treatment, and to the fact that some breast cancer survivors may report treatment-related side effects that last up to several years after the end of active treatment. Among these effects, cancer-related fatigue and cognitive disorders have been clearly identified as very prevalent in certain subgroups of breast cancer survivors and can be extremely distressing.

The most robust evidence links inflammatory processes, which can be triggered or worsened by cancer treatment, to the onset and persistence of fatigue and cognitive impairment. Accordingly, several types of interventions have proven to be beneficial in reducing these symptoms by acting on pro-inflammatory mediators, particularly interventions aimed at increasing physical activity. At the same time, although there has been progress in the definition of the pathophysiology of cancer-related fatigue and cognitive impairment, particularly with the use of neuroimaging and biological data, complementary studies are needed to better elucidate the underlying mechanisms and predictors of these persistent symptoms. More research is warranted to look further away from completion of treatment and up to years into the survivorship period, in order to evaluate patients with sufficient follow-up time for studies to be informative about the long-term impact of interventions on persistence of symptoms. In addition, larger and adequately powered trials and assessment of combination of programs are encouraged.

Despite the acknowledged urgency to address late and long-term survivorship issues among patients with breast cancer, it has often been remarked that there is still a lack of appropriate identification and correct management of long-term symptoms in clinical practice, and that there is a substantial risk that problems like fatigue and cognitive difficulties remain under-diagnosed or under-reported, and therefore under-treated. Consensus exists among major cancer societies that cancer-related fatigue and cognitive problems should be managed in a multidisciplinary setting and with a comprehensive approach. This includes providing specific counseling and being able to refer patients to professionals with adequate expertise to address such symptoms, including physical therapists, psychologists, behavioral therapists, nutritionists, and social workers. Although awareness about these issues has now increased, more education, training of healthcare professionals, as well as improved consistency of practice across institutions and involvement of policy-makers is needed, not only to improve cancer-related fatigue and cognitive difficulties, but also to avoid many other long-term effects of cancer treatment among breast cancer survivors. 

## Figures and Tables

**Table 1:** Validated instruments to assess self-reported fatigue [5,6,8].

Instruments to Assess Fatigue
Breast Cancer Survivor Symptom Survey fatigue subscale Brief Fatigue Inventory European Organization for Research and Treatment of Cancer–Quality-of-Life Questionnaire (EORTC)-C30 fatigue subscale EORTC Quality of Life Module Measuring Cancer Related Fatigue (EORTC QLQ-FA12) Fatigue Symptom Inventory Memorial Symptoms Assessment Scale Short Form Piper Fatigue Scale-revised Somatic and Psychological Health Report fatigue scale

**Table 2:** Interventions to reduce fatigue among survivors of breast cancer post-treatment.

Non-Pharmacologic
Counseling/education (delivered in person or remotely) Physical activity (maintaining recommended levels of activity, i.e., 150 minutes of moderate intensity activity/week, combining endurance and resistance training; beware of safety issues, personalize activity levels if needed; consider physical therapy and rehabilitation if needed) Other physical and mind–body interventions Yoga Massage therapy Acupuncture Music therapy Mindfulness meditation and relaxation Reiki Qigong Psychosocial interventions Psycho-educational therapies Cognitive-behavioral therapy Treatment for emotional distress Sleep hygiene
**Pharmacologic**
Supplements (ginseng, vitamin D)

Note: Not all listed interventions have the same level of evidence and indications in the treatment of cancer-related fatigue. Refer to the text for specific settings, patient population, and strength of available evidence regarding each specific intervention. Consider also adjusting treatments for concomitant symptoms such as pain, address existing comorbidities, and assess and improve nutrition if needed.

**Table 3:** Interventions to reduce cancer-related cognitive impairment among breast cancer survivors.

Publication	Study Design	Intervention	Participants	Outcomes	Tool	Results	Adverse Events	Conclusion
**Cognitive Rehabilitation Intervention**								
Von Ah et al. [108]	3-arms RCT	6–8 week of memory (A) or processing (B) intervention or WL (C)	82 breast cancer survivors ≥1 year post-CT (26 memory training, 27 speed training, 27 WL)	Objective cognition Subjective cognition QoL Fatigue	RAVLT FACT-Cog SF-36 FACT-F	A and B > C A and B > C B > C B > C	Not reported	Improvements in cognitive function, fatigue, and QoL.
Ercoli et al. [109]	RCT	5-week CR or WL	48 breast cancer survivors 18 months–5 years post-CT (32 CR, 16 WL)	Objective cognition Subjective cognition	RAVLT PAOFI	*p* = 0.02 *p* = 0.01	Not reported	Improvements in cognitive complaints and memory scores.
Damholdt et al. [110]	RCT	6-week web-based CR or WL	157 breast cancer survivors (94 CR, 63 WL)	Objective cognition Subjective cognition	PASAT CFQ	NS NS	Not reported	NS impact on cognitive function.
Bray et al. [111]	RCT	15-week web-based CR or WL	242 cancer survivors, 89% breast cancer, 6–60 months post-adjuvant CT (122 CR, 121 WL)	Objective cognition Subjective cognition QoL Fatigue	Cogstate FACT-Cog PCI FACT-G FACT-F	NS *p* < 0.001 Better score Better score	Not reported	Improvements in cognitive complaints, QoL, and fatigue.
Mihuta et al. [112]	RCT	4-week web-based CR or WL	65 cancer survivors, 97% breast cancer, >6 months post-CT	Objective cognition Subjective cognition	ReCog FACT-Cog PCI	NS *p* = 0.089	Not reported	Improvement trends in cognitive complaints.
**Physical Activity or Relaxation Program Interventions**								
Campbell et al. [113]	RCT	24-week aerobic exercise or control	19 breast cancer, 3 months–3 years post-adjuvant CT (10 exercise, 9 control)	Objective cognition Subjective cognition	TMT-A FACT-Cog	*p* < 0.01 NS	Not reported	Processing speed improved but not cognitive complaints.
Hartman et al. [114]	RCT	12-week physical activity or control	87 breast cancer survivors with CT completion (43 exercise, 44 control)	Objective cognition Subjective cognition	NIH Toolbox PROMIS	NS NS	Not reported	NS impact on cognitive function.
Northey et al. [115]	3-arms RCT	12-week HIIT, MOD, or WL	17 breast cancer survivors <2 years post-diagnosis (6 HIIT, 5 MOD, 6 WL)	Objective cognition Cerebrovascular function	CogState MCAVmean	NS NS	Not reported	NS impact on cognitive and cerebrovascular outcomes.
Derry et al. [116]	RCT	12-week yoga intervention or WL	200 breast cancer survivors, 2 months–3 years post-adjuvant CT (100 yoga, 100 placebo)	Subjective cognition	BCPT Cognitive Problems Scale	*p* = 0.003 3 months after yoga	Not reported	Improvements in cognitive complaints at 3-month follow-up.
Myers et al. [117]	3-arms RCT	8-week qigong (A), gentle exercise (B), or survivors support (C)	50 breast cancer survivors, 2 months–8 years post-adjuvant CT (19 qigong, 20 exercise, 9 support)	Objective cognition Subjective cognition	TMT-A RAVLT FACT-Cog PCI	A > B NS A > C *p* = 0.01	Not reported	Improvements in cognitive complaints and processing speed.
**Pharmacologic Interventions**								
Mar Fan et al. [118]	Double-blind RCT	Dexmethylphenidate until CT completion or placebo	57 breast cancer (29 experimental, 28 placebo) under adjuvant CT	Objective cognition QoL Fatigue	HSCS HVLT-R FACT-G FACT-F	NS NS NS NS	Higher with more events that led to discontinuation	NS impact on cognition, QoL, or fatigue and more adverse events.
Lower et al. [119]	Double-blind RCT	Dexmethylphenidate 8 weeks or placebo	154 cancer patients, 75% breast cancer (76 experimental, 78 placebo) CT completion ≥2 months	Objective cognition Fatigue	HSCS SNAP FACIT-F	NS NS *p* = 0.02	Higher with more events that led to discontinuation	NS impact on cognitive function and more adverse events.
Kohli et al. [120]	RCT	Modanil 4 weeks or placebo if positive response after 4 previous weeks	68 breast cancer survivors (34 modanil, 34 placebo)	Objective cognition	CDR computerized assessment	NS	Not reported	NS impact on cognitive function.
Lawrence et al. [121]	Double-blind RCT	Donepezil 24 weeks or placebo	62 breast cancer survivors, adjuvant CT 1–5 years prior, with cognitive dysfunction (31 donepezil, 31 placebo)	Objective cognition Subjective cognition QoL Fatigue	HVLT-R FACT-Cog SF-36 FACIT-F	*p* = 0.03 NS NS NS	Not reported	NS impact on cognitive complaint, QoL, and fatigue.
O’Shaughnessy et al. [122]	Double-blind RCT	Epoetin alpha 12 weeks or placebo	94 breast cancer with adjuvant or neoadjuvant CT (47 epoetin, 47 placebo)	Objective cognition QoL Fatigue	EXIT25 LASA FACT-An	NS Better score Better score	1 death with epoetin alfa (CVA)	NS impact on cognitive function. Improvement of fatigue and QoL.

Abbreviations: RCT: Randomized Controlled Trial; WL: Wait-list control; CT: Chemotherapy; QoL: Quality of life; RAVLT: Rey Auditory Verbal Learning Test; FACT-Cog: Functional Assessment of Cancer Therapy-Cognition; SF-36: 36-Item Short Form Health Survey; FACT-F: Functional Assessment of Cancer Therapy-Fatigue; CR: Cognitive rehabilitation; PAOFI: Patient’s Assessment of Own Functioning Inventory; PASAT: Paced Auditory Serial Addition Test; NS: Non-significant; CFQ: Cognitive Failures Questionnaire; PCI: Perceived cognitive impairment; FACT-G: Functional Assessment of Cancer Therapy-General; ReCog: Responding to Cognitive Concerns; TMT: Trail making test; NIH: The National Institutes of Health; PROMIS: Patient-Reported Outcomes Measurement Information System; HIIT: High-intensity interval training; MOD: Moderate-intensity continuous training; MCAVmean: Mean flow velocity in the right middle cerebral artery; BCPT: Breast Cancer Prevention Trial; HSCS: High Sensitivity Cognitive Screen; HVLT-R: Revised Hopkins Verbal Learning Test; SNAP: Modified Swanson, Nelson and Pelham Attention Deficit/Hyperactivity Scale; FACIT-F: Functional Assessment of Chronic Illness Therapy-Fatigue; CDR: Cognitive Drug Research; EXIT25: Executive Interview; LASA: Linear Analog Scale Assessment; FACT-An: Functional Assessment of Cancer Therapy-Anemia; CVA: Cerebrovascular accident.
